# Supplementary material for: Introducing the crystalline phase of dicalcium phosphate monohydrate
Source: Nat Commun. 2020 Mar 24;11:1546. doi: 10.1038/s41467-020-15333-6 (PMC7093545; doi:10.1038/s41467-020-15333-6)
Supplement: Supplementary file 1 — Supplementary Information [file 41467_2020_15333_MOESM1_ESM.pdf]

Supplementary Information

Introducing the Crystalline Phase of Dicalcium Phosphate Monohydrate

Lu et al.

Contents

1. FIGURES 2

2. TABLES 12

3. SUPPLEMENTARY COMMENT SECTION 18

4. REFERENCES 19

1. Figures

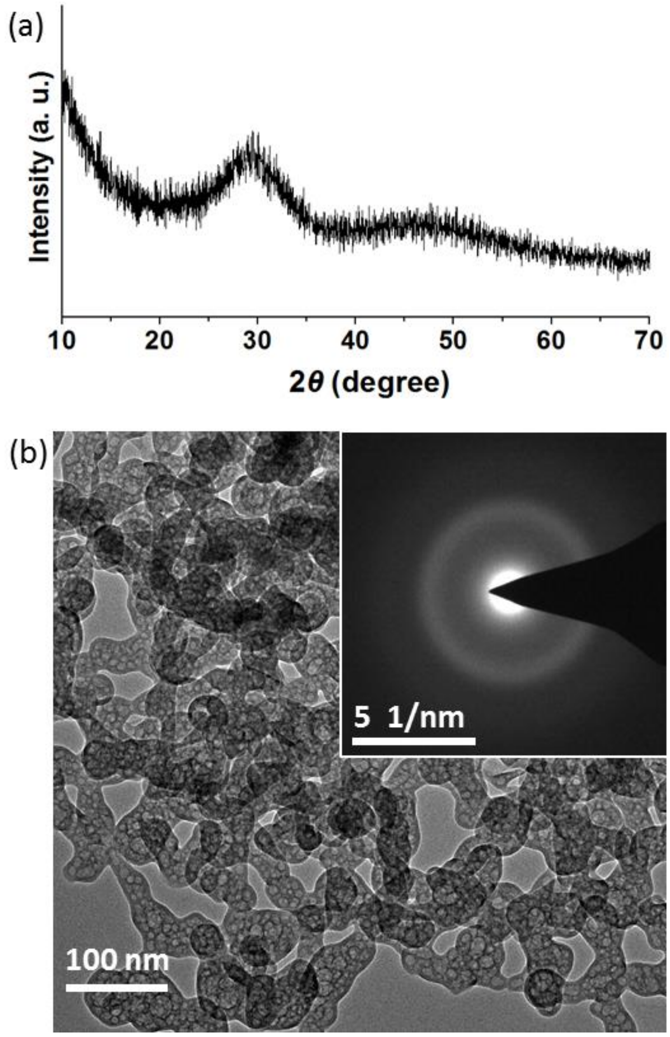

Supplementary Figure 1. XRPD pattern (a) and TEM image (b) with ED pattern (inset) of the as-prepared ACHP.

The broad feature at 2θ = 25-35° on XRPD pattern (a), and the absence of distinct reflections on the ED (inset) pattern confirm the amorphous character of ACHP.

The images of the ACHP (b) show spherical and aggregated nanoparticles of sizes between tens-hundreds of nanometers, which is also the typical ACP morphology (*1*). Source data are provided as a Source Data file.

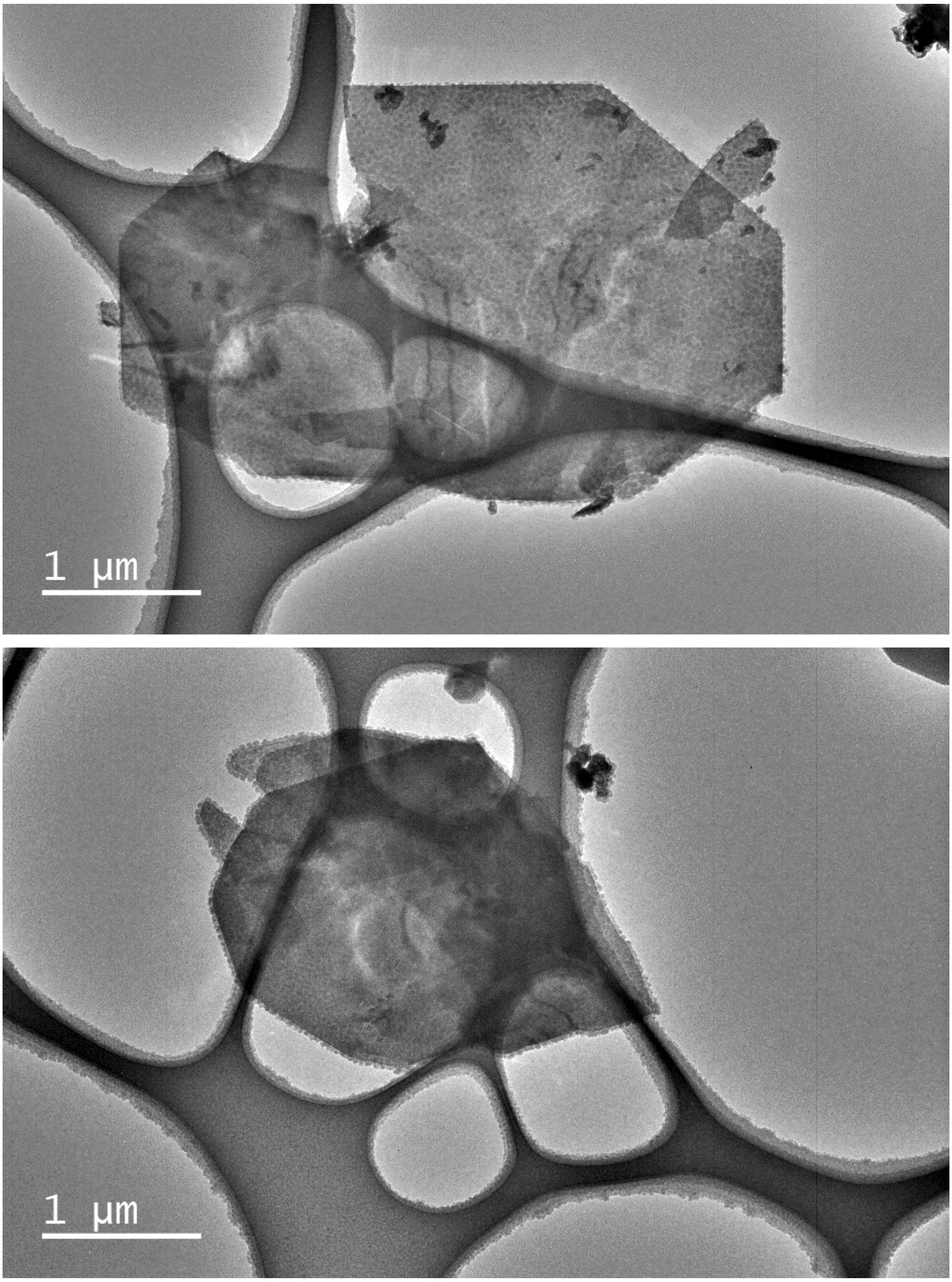

Supplementary Figure 2. Crystals of DCPM prepared under mixed solvent conditions show a thin plate-like morphology with facetted edges. The small crystals at the edge of the DCPM crystals are ice formed during liquid nitrogen cooling in the TEM. Source data are provided as a Source Data file.

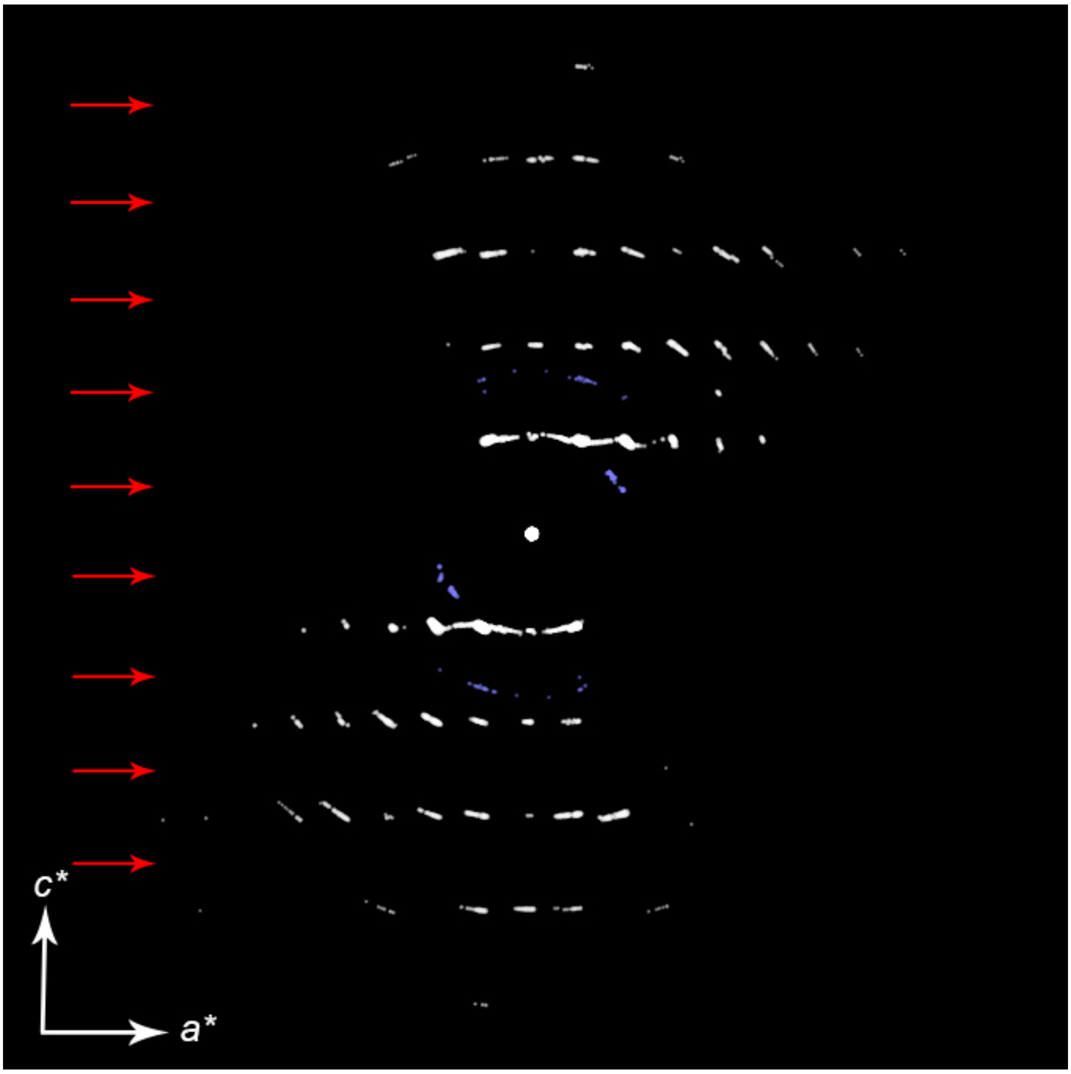

Supplementary Figure 3. A section through the reconstructed reciprocal lattice of a cRED data set from DCPM including the *h0l* family of reflections. In this data set reflections with odd *l*-indices are very close to extinct. This is consistent with a *c*-glide in the crystal structure of DCPM. Horizontal lines with odd *l*-indices are marked by red arrows. Note that traces of diffraction from small ice crystals formed during the data collection at liquid nitrogen temperature can be observed (colored in light blue).

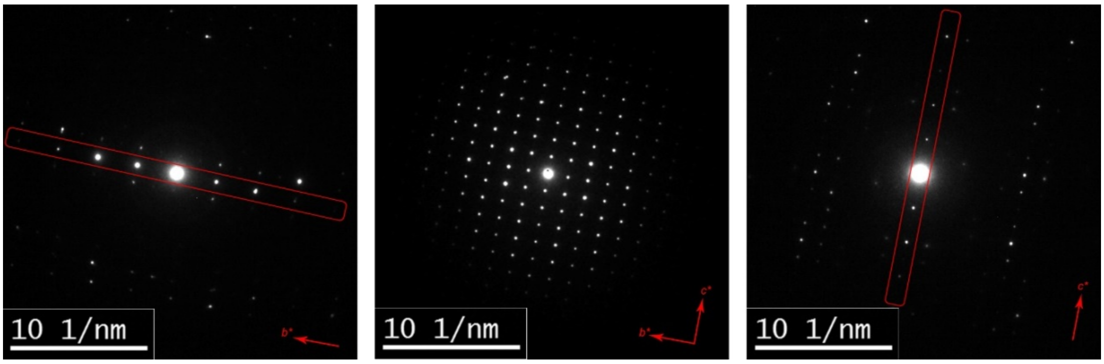

Supplementary Figure 4. Three selected area electron diffraction patterns from a crystal of DCPM were acquired to further illustrate the systematic extinctions. The middle pattern is acquired along the [100] direction and affected by dynamic scattering. The pattern to the left is tilted away from the [100] direction by ~8° around the *b**-axis. It is clear that systematic extinctions appear along the *b**-axis as reflections with odd *k*-indices are now absent. Following the parallel procedure, the pattern to the right reveals clear extinctions for reflections in the *00l* family with odd *l*-indices when the crystal is tilted around the *c**-axis by ~8°. This is clearly consistent with the symmetry $P2_1/c$. Source data are provided as a Source Data file.

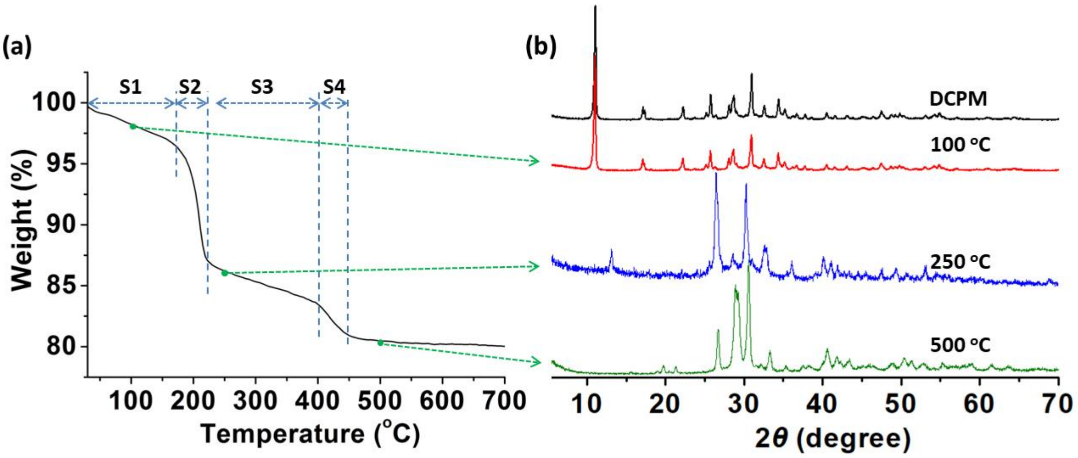

Supplementary Figure 5. (a) Thermogravimetric analysis (TGA) of DCPM. (b) XRPD patterns of DCPM after heating at different temperature for 30 min. The symbols + denote DCP; and # calcium pyrophosphate. TGA shows that DCPM loses water in four steps: S1, S2, S3 and S4. The XRPD pattern of the sample heated at 100 ºC reveals no significant difference compared to that before heating, indicating S1 is a step of water desorption. The XRPD pattern of sample heated at 500 ºC shows that DCPM changes to calcium pyrophosphate ultimately. Using the TGA and ICP-OES data (Ca/P atomic ratio 1.01), a formula of $[Ca_{1.5}(PO_4)][CaHPO_4]_{49}\cdot 48H_2O$, that is, to within a very good approximation, $CaHPO_4\cdot H_2O$, is obtained. A sample prepared in mixed solvents was used here. Source data are provided as a Source Data file.

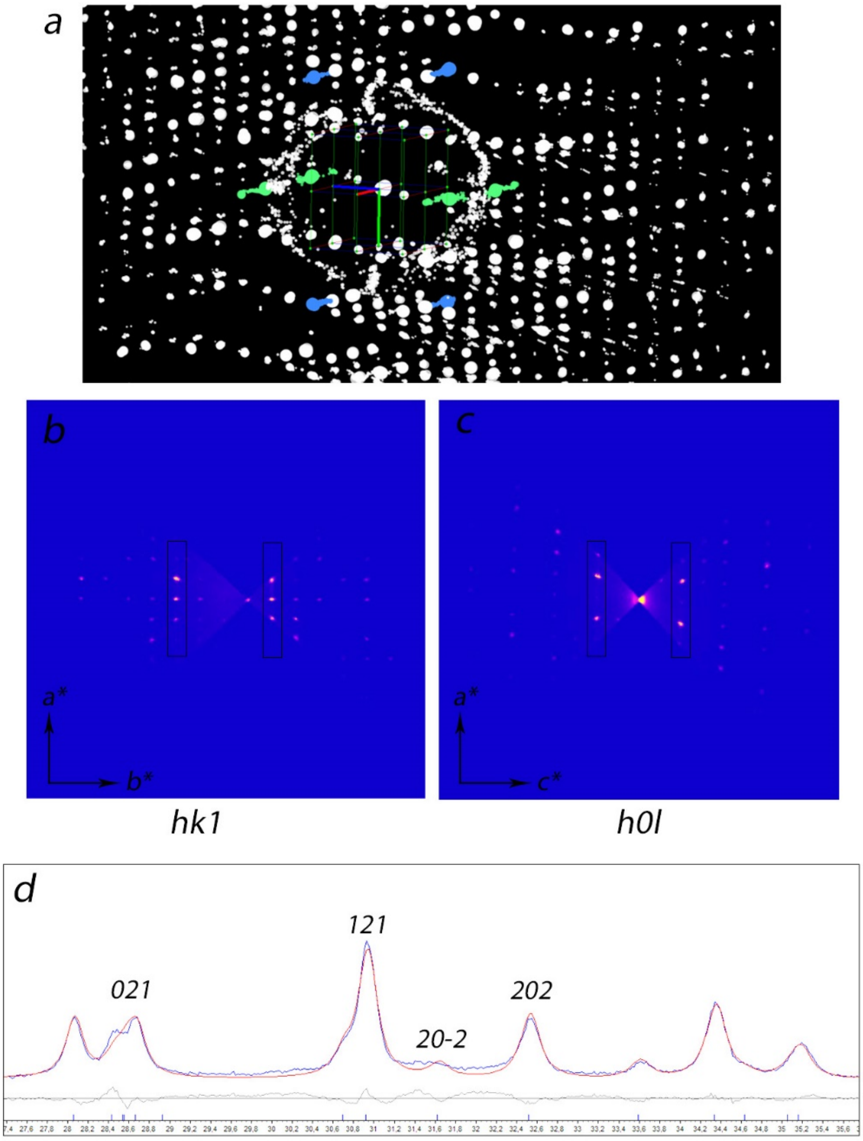

Supplementary Figure 6. (a) Part of the reconstructed three-dimensional reciprocal lattice from data collected from DCPM shows some diffusely scattered streaks running along the *a**-axis. The elliptical features arise due to scattering from small ice crystals formed when the sample was cooled during data collection. The *121* reflection and its symmetry equivalents (blue) show diffuse intensities extending along the *a**-axis to both higher and lower scattering angles. The *h02* lines also exhibit diffuse intensities along the *a**-axis. (b and c) Sections through a full voxel-by-voxel reconstruction reveals weak diffuse scattering around the *121* reflection in the *hk*1 section (a), some weak diffuse scattering is also present along the *h*02 line in (c). (d) Enlarged section of the XRPD pattern of DCPM between 27.4° and 35.7°. In this regime, some diffuse intensities are present which cannot be fitted well with the pseudo-Voigt peak shape. The corresponding d-spacings, as well as indices, for the reflections are consistent with the diffuse scatting present in the cRED data.

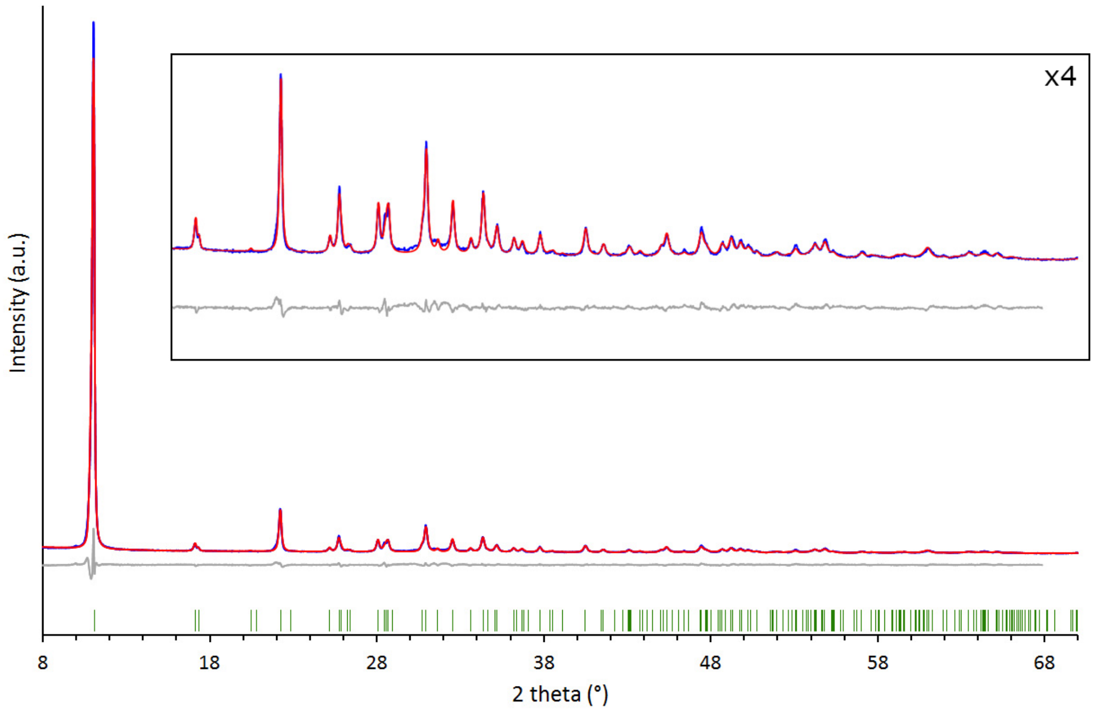

Supplementary Figure 7. Rietveld refinement plot of DCPM prepared from mixed solvent. The observed, calculated and difference X-ray powder diffraction patterns are shown in blue, red and grey, respectively. Tick marks indicated the positions of Bragg reflections. The data was collected utilizing Cu Kα radiation (λ=1.54178 Å). The fit converges well with a residual R_{wp} of 6.667%. The range 17°-70° is enlarged in the inset for clarity. In the domain 29° – 34° there are some issues with the peak shape of some reflections. This can be attributed the presence of some diffuse scattering, which is also observed in cRED data. This is due to some stacking disorder present in the material introducing imperfections in the connectivity of the layers. Source data are provided as a Source Data file.

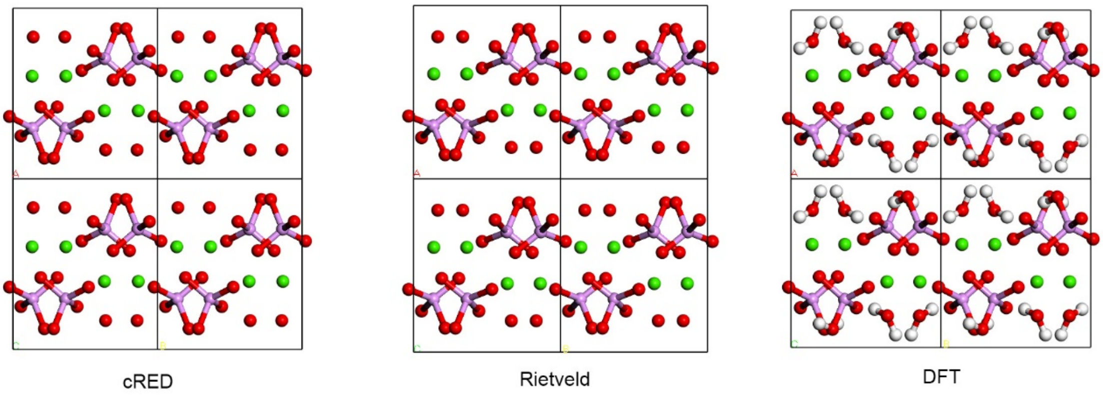

Supplementary Figure 8. Comparison of the structures of DCPM after refinement against cRED data (left), XRPD data (middle) as well as the DFT optimized structure (right). The structures are viewed along the [001] direction. Green represents Ca, purple P, red O and white H.

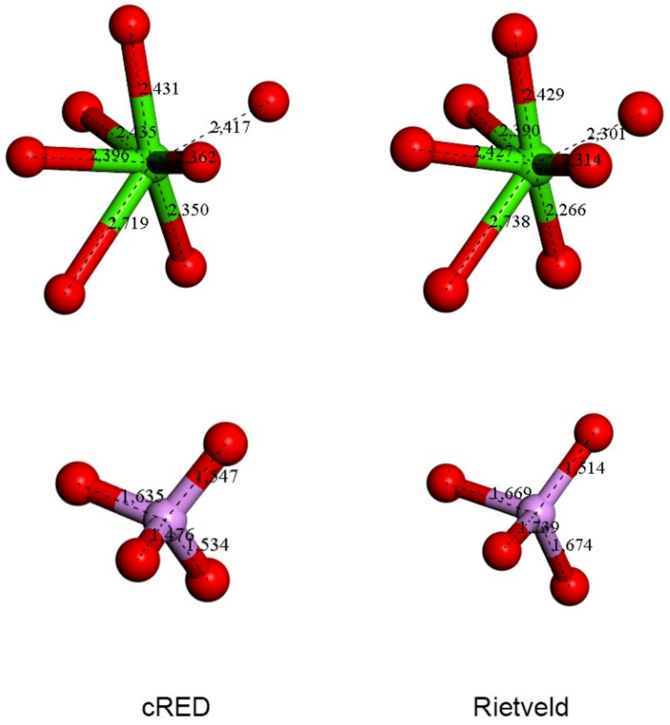

Supplementary Figure 9. Comparison of the coordination around the calcium ion and phosphorous atom for the structures after refinement against cRED (left) and XRPD (right) data. Green represents Ca, purple P and red O. The oxygen atom to the top right of the Ca atom is the water molecule in the vicinity of the Ca ion. Bond lengths and distances are indicated in Ångströms.

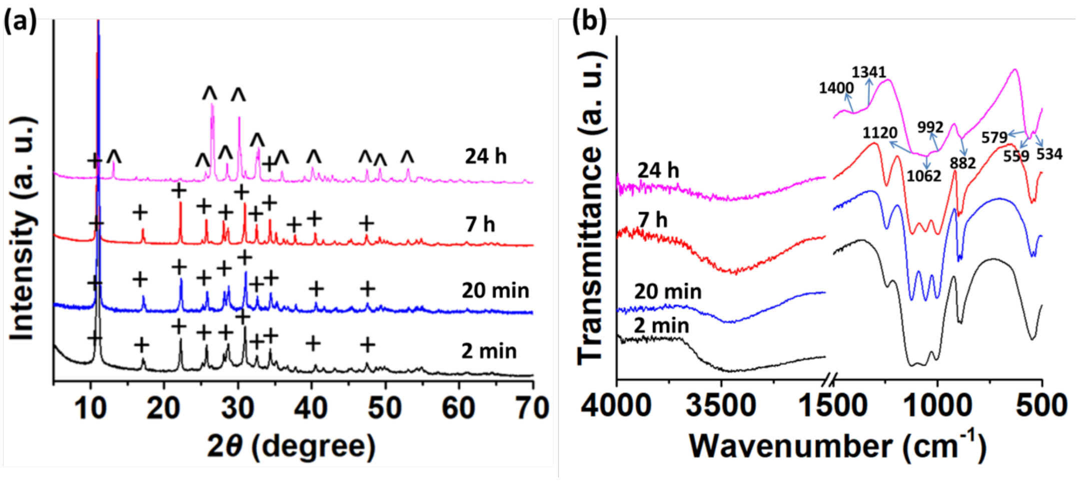

Supplementary Figure 10. XRPD patterns (a) and IR spectra (b) of the phase evolution during the DCPM preparation in mixtures of methanol and water. (a) The symbols + and ^ mark the diffraction peaks of DCPM and DCP, respectively. (b) The samples of 2 min − 7 h show typical spectra of DCPM despite the varied intensities of the bands. That of 24 h present bands of DCP at 1400, 1341, 1120, 1062, 992, 882, 579, 559, 534 cm^{-1}. Source data are provided as a Source Data file.

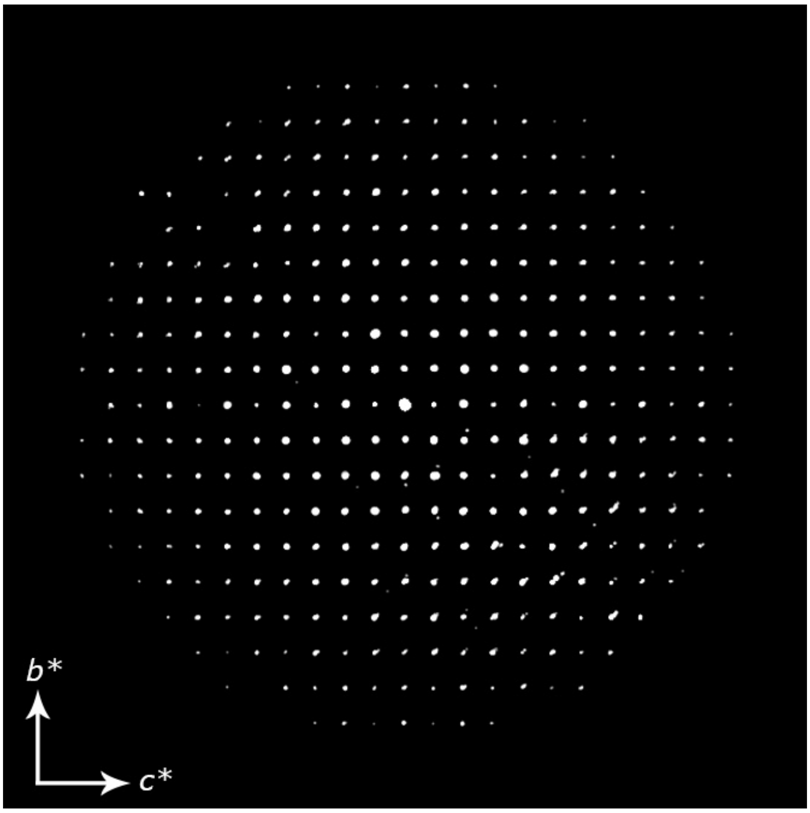

Supplementary Figure 11. Reconstructed reciprocal lattice from cRED data obtained from DCPM prepared in humid air. Ab-initio structure determination resulted in the same structure as for the specimen prepared under mixed solvent conditions.

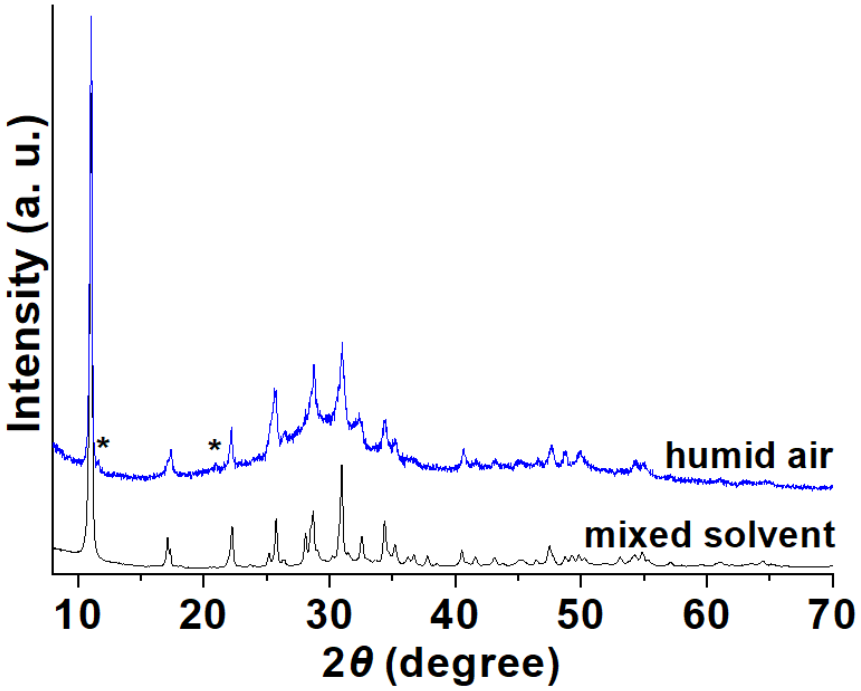

Supplementary Figure 12. The XRPD patterns of DCPM prepared from ACHP in humid air and mixed solvent as indicated. The asterisk marks reflections due to impurities of DCPD. Source data are provided as a Source Data file.

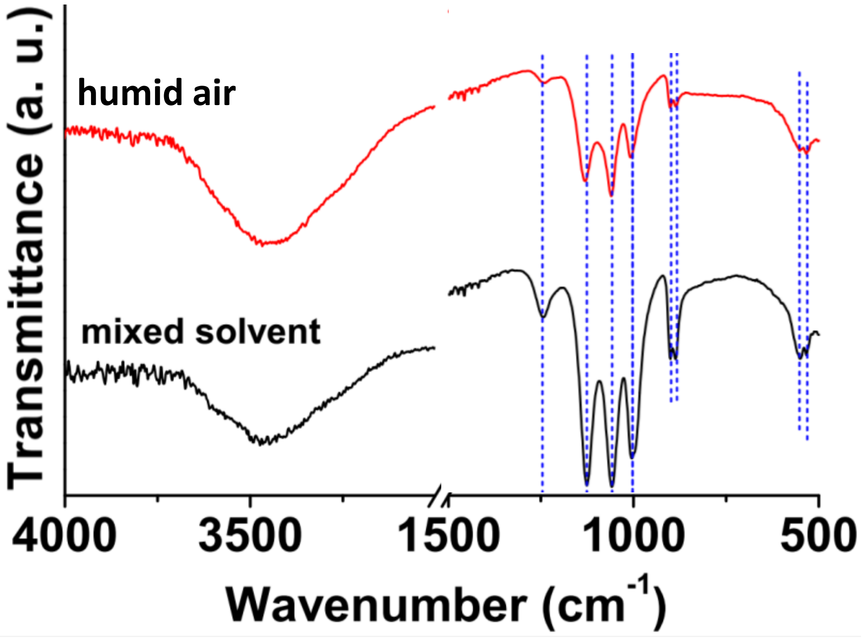

Supplementary Figure 13. The FTIR spectra of DCPM prepared from ACHP in humid air and mixed solvent. The dashed vertical lines are guides for the eye to illustrate the positions of the bands. Source data are provided as a Source Data file.

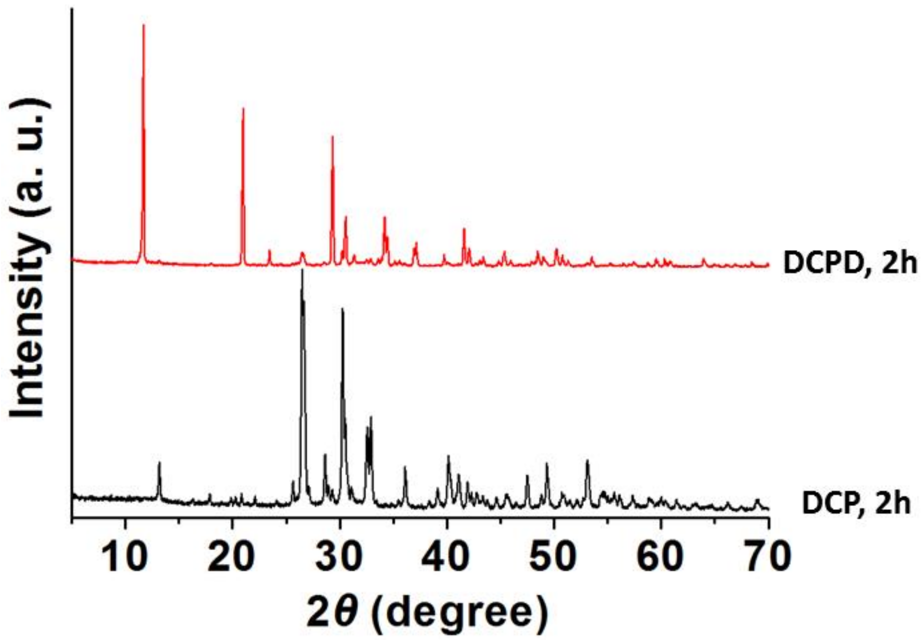

Supplementary Figure 14. XRPD patterns of particles retrieved after dispersing DCP and DCPD in water for 2 h. Source data are provided as a Source Data file.

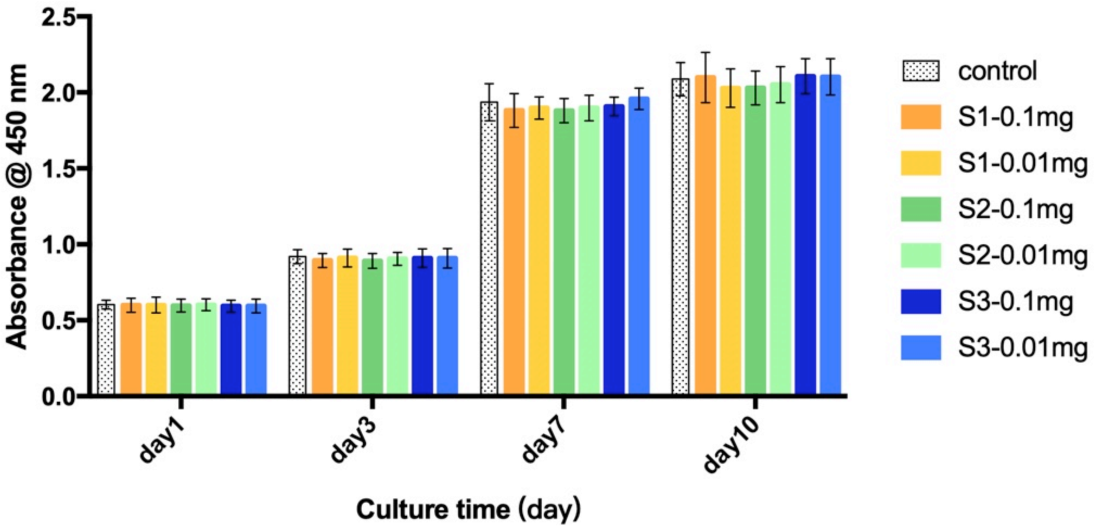

Supplementary Figure 15. The cell viability in presence of DCP (S1), DCPM (S2) and DCPD (S3) evaluated using the CCK-8 test, in which the absorbance at 450 nm is positively correlated to cell viability. The colors of columns represent different samples with certain amounts of CaPs added into each well of a 24 well-plate for co-incubating with cells. In this figure, from day1- day10, the cell viability of DCPM is nearly the same as DCP and DCPD, indicating they have similar biocompatibility in vitro. Error bars illustrate to the standard deviation (N=6). Source data are provided as a Source Data file.

2. Tables

Supplementary Table 1. Statistics for the refinement from continuous rotation electron diffraction data.

Data statistics for refinement on cRED data*

Crystal system	Monoclinic
Space group	$P2_1/c$ (No. 14)
a, Å	8.0063(4)
b, Å	6.7954(5)
c, Å	7.7904(5)
α, º	90
β, º	91.548(4)
γ, º	90
Volume, Å³	423.63(5)
λ, Å	0.0251
Exposure time per frame, s	0.5
Tilt range, º	102.76
Integration range, °/frame	0.574
Completeness, %	59.7
I/σ	3.77
Resolution	0.80
R_{int}	0.183
No. of symmetry independent reflections	563
No. of parameters	29
Refinement R1	0.260

* Unit cell parameters as determined from XRPD data were used for the final refinement.

Supplementary Table 2. Statistics for the Rietveld refinement of DCPM prepared in a mixed solvent.

Data statistics	
Crystal system	Monoclinic
Space group	$P2_1/c$ (No. 14)
a, Å	8.0063(4)
b, Å	6.7954(5)
c, Å	7.7904(5)
α, º	90
β, º	91.548(4)
γ, º	90
Volume, Å³	423.69(5)
λ, Å	1.54178
No. of parameters	56
R_p	0.05068
R_{wp}	0.0667

Supplementary Table 3. Comparison of the crystal parameters of CaPs (*2, 3*).

**Compound**	**Formula**	**Ca/P ratio**	**Mineral**	**Space group**	**Unit cell parameters**
Dicalcium phosphate (DCP)	$CaHPO_4$	1	Monetite	Triclinic $P\bar{1}$	a=6.910(1) Å<br>b=6.627(2) Å<br>c=6.998(2) Å<br>α=96.34(2)°<br>β=103.82(2)°<br>γ=88.33(2)°
Dicalcium phosphate monohydrate (DCPM)	$CaHPO_4 \cdot H_2O$	1		Monoclinic $P2_1/c$	a=8.0062(4) Å<br>b=6.7954(5) Å<br>c=7.7904(5) Å<br>α=γ=90°<br>β=91.548(4)
Dicalcium phosphate dehydrate (DCPD)	$CaHPO_4 \cdot 2H_2O$	1	Brushite	Monoclinic Ia	a=5.812(2) Å<br>b=15.180(3) Å<br>c=6.239(2) Å<br>α=γ=90°,<br>β=116.42(3)°
Octacalcium phosphate (OCP)	$Ca_8H_2(PO4)_6 \cdot 5H_2O$	1.33		Triclinic $P\bar{1}$	a=19.692(4) Å<br>b=9.523(2) Å<br>c=6.835(2) Å<br>α=90.15(2)°<br>β=92.54(2)°<br>γ=108.65(1)°
α-Tricalcium phosphate (α-TCP)	$Ca_3(PO4)_2$	1.5		Monoclinic $P2_1/a$	a=12.887(2) Å<br>b=27.280(4) Å<br>c=15.219(2) Å<br>β=126.20(1)°
β-Tricalcium phosphate (β-TCP)	$Ca_3(PO_4)_2$	1.5		Rhombohedral $R3Ch$	a=b=10.4183(5)Å<br>c=37.3464(23) Å<br>γ=20°
Hydroxyapatite (HAP)	$Ca_{10}(PO_4)_6(OH)_2$	1.67	Hydroxyapatite	Monoclinic $P2_1/b$<br><br>hexagonal $P6_3/m$	a=9.84214(8)<br>b=2a<br>c=6.8814(7) Å<br>γ=120°;<br><br>a=b= 9.4302(5),<br>c=6.8911(2) Å<br>γ=120°

Supplementary Table 4. Unit cell parameters and thermodynamic properties of monetite, brushite and DCPM as computed from first principles (QM) geometry optimization. For comparison, the results of *ab initio* MD at 330 K and force field (FF) calculations are also shown for DCPM. Here ΔG(H_2O) represents the estimated free energy of water incorporation relative to monetite at standard conditions. Values in parenthesis are the percentage errors in cell parameters relative to the experimental values. As expected, the QM optimizations generally show systematic errors of less than 1%, except for the a and β lattice parameters of DCPM which reflects the greater difficulty of accurately describing layered structures. Similarly, the absolute free energies for water incorporation to form mineral hydrates are often overestimated by most exchange-correlation functionals(*4*), as is the case here, where the free energy difference to convert monetite to brushite is +0.9 kJ mol^{-1}.

	Monetite	Brushite	DCPM (QM)	DCPM (FF)	DCPM (MD)
a (Å)	6.9370 (0.3)	5.8264 (0.5)	7.7324 (-3.4)	8.4178 (5.2)	7.779 (-2.8)
b (Å)	6.6540 (0.5)	14.9863 (-0.9)	6.8730 (1.2)	6.7651 (-0.4)	6.949 (2.3)
c (Å)	6.9270 (-0.3)	6.1401 (-0.7)	7.7193 (-0.8)	7.8368 (0.7)	7.959 (2.2)
α (°)	96.02 (-0.2)	90.00 (0.0)	90.00 (0.0)	90.00 (0.0)	90.06 (0.07)
β (°)	103.51 (-0.3)	115.19 (-1.0)	92.93 (1.5)	86.49 (-5.5)	94.51 (3.3)
γ (°)	88.21 (-0.1)	90.00 (0.0)	90.00 (0.0)	90.00 (0.0)	90.05 (0.06)
ΔG(H_2O) (kJ mol^{-1})	N/A	+10.1	+9.9	+11.7	-

Supplementary Table 5. Comparison of DCPM with the chemical properties of other CaPs.

**Compound**	**Transformation products by heating**	**Transformation products in aqueous solutions**
Dicalcium phosphate (DCP)	calcium pyrophosphate (CPP)<br>Ref (*5*)	HAP,<br>OCP,<br>Ref (*6*)
Dicalcium phosphate monohydrate (DCPM)	DCP, CPP	HAP
Dicalcium phosphate dehydrate (DCPD)	DCP, CPP<br>Ref (*5*)	OCP[b],<br>HAP<br>Ref (*7, 8*)
Octacalcium phosphate (OCP)	collapsed OCP, DCP, CPP<br>HAP, β-TCP<br>Ref (*7, 9*)	HAP<br>Ref (*9*)
α-Tricalcium phosphate (α-TCP)		OCP[ad], HAP<br>Ref (*10, 11*)
β-Tricalcium phosphate (β-TCP)	α-TCP<br>Ref (*12*)	HAP[t]<br>Ref (*13*)
Hydroxyapatite (HAP)	Oxyhydroxyapatite (OHAP),<br>α –TCP,<br>tetracalcium phosphate (TTCP)<br>Ref (*14*)	DCPD[a],<br>Whitlockite[a]<br>Ref (*15*)

HAP contains calcium-deficient hydroxyapatite (CDHA).

[a]: acid environment

[b]: base environment

[ad]: with substitution or additives

[t]: high temperature (>100 ^{o}C)

Supplementary Table 6. Adsorption capacity of DCP, DCPM and DCPD for organic molecules.

	DCP (g/g)	DCPM (g/g)	DCPD (g/g)
**Methyl blue (in water)**	0.52±0.01	1.32±0.15	0.20±0.01
**Methyl blue (in methanol)**	0.17±0.02	0.36±0.10	0.075±0.021
**Congo red (in water)**	undetected	0.11±0.02	0.033±0.013
**Doxorubicin hydrochloride (in water)**	0.075±0.020	0.14±0.02	0.045±0.012
**Ibuprofen (in ethanol)**	undetected	0.13±0.03	undetected

The adsorption capacity was determined as the ratio adsorbate/adsorbent (g/g), and values shown correspond to the mean ± standard deviation (N=3).
Undetected: <0.010 g/g.

3. Supplementary Comment Section

About the space group $P2_1/c$ of DCPM

While the data does contain multiple scattering, all our results suggest that the $P2_1/c$ symmetry is the proper choice. By acquiring electron diffraction patterns slightly tilted away from zone axis the multiple scattering will be reduced and the systematic extinctions will show up clearly. In Supplementary Figure 3, electron diffraction patterns have been acquired along the [100] direction, as well as tilted ~8° away from the main zone axis around the b^* and c^*-axes. While the pattern acquired along the [100] direction is heavily affected by multiple scattering (where no extinctions are visible), after tilting the crystal ~8° from the zone axis, clear extinctions show up both along the b^* and c^*-axes. Among the primitive monoclinic space groups there is just one extinction symbol which is consistent with extinctions along two perpendicular axes leaving us with just one unique choice of symmetry for DCPM, namely $P2_1/c$ (No. 14). The validity of the space group is also supported by quantum mechanical calculations that find no imaginary phonon modes for the structure optimized within this symmetry.

Since the morphology of the DCPM crystals is plate-like, the crystals will likely pass close to the *0kl* plane of the reciprocal lattice and hence the cRED diffraction data will be influenced by dynamic effects.

Evaluation of several cRED data sets shows that reflections belonging to the *h*03 and *h01* families appear with different intensities. For data sets that do not pass close to the main zone axes, e.g. the data set shown in Supplementary Figure 4, the given reflections appear absent or very weak. This is clearly consistent with a *c*-glide in the structure of DCPM. This shows that multiple scattering is responsible for the observed intensities appearing in Figure 1c of the main article showing the *h*0*l* family of reflections.

4. References

1. S. V. Dorozhkin, Amorphous calcium (ortho)phosphates. *Acta Biomater.* **6**, 4457-4475 (2010).
2. L. J. Wang, G. H. Nancollas, Calcium Orthophosphates: Crystallization and Dissolution. *Chem. Rev.* **108**, 4628-4669 (2008).
3. S. V. Dorozhkin, Calcium Orthophosphates in Nature, Biology and Medicine. *Materials* **2**, 399-498 (2009).
4. R. Demichelis, P. Raiteri, J. D. Gale, R. Dovesi, Examining the Accuracy of Density Functional Theory for Predicting the Thermodynamics of Water Incorporation into Minerals: The Hydrates of Calcium Carbonate. *J. Phys. Chem. C* **117**, 17814-17823 (2013).
5. A. Dosen, R. F. Giese, Thermal decomposition of brushite, CaHPO4 center dot 2H(2)O to monetite CaHPO4 and the formation of an amorphous phase. *Am. Mineral.* **96**, 368-373 (2011).
6. A. Lebugle, B. Sallek, A. T. Tai, Surface modification of monetite in water at 37 degrees C: characterisation by XPS. *J. Mater. Chem.* **9**, 2511-2515 (1999).
7. W. E. Brown, J. R. Lehr, J. P. Smith, A. W. Frazier, Crystallography of Octacalcium Phosphate. *J. Am. Chem. Soc.* **79**, 5318-5319 (1957).
8. E. J. Duff, Orthophosphates. Part III. The hydrolysis of secondary calcium orthophosphates. *J. Chem. Soc. A*, 917-921 (1971).
9. D. G. A. Nelson, J. D. Mclean, High-Resolution Electron-Microscopy of Octacalcium Phosphate and Its Hydrolysis Products. *Calcif. Tissue Int.* **36**, 219-232 (1984).
10. A. Bigi, E. Boanini, R. Botter, S. Panzavolta, K. Rubini, α-Tricalcium phosphate hydrolysis to octacalcium phosphate: effect of sodium polyacrylate. *Biomaterials* **23**, 1849-1854 (2002).
11. C. Durucan, P. W. Brown, α-Tricalcium phosphate hydrolysis to hydroxyapatite at and near physiological temperature. *J. Mater. Sci. Mater. Med.* **11**, 365-371 (2000).
12. J. H. Welch, W. Gutt, 874. High-temperature studies of the system calcium oxide–phosphorus pentoxide. *J. Chem. Soc. (Resumed)*, 4442-4444 (1961).
13. T. Goto, Y. Kim, K. Kikuta, C. Ohtsuki, Comparative study of hydroxyapatite formation from α- and β-tricalcium phosphates under hydrothermal conditions. *J. Ceram. Soc. Jpn.* **120**, 131-137 (2012).
14. C.-J. Liao, F.-H. Lin, K.-S. Chen, J.-S. Sun, Thermal decomposition and reconstitution of hydroxyapatite in air atmosphere. *Biomaterials* **20**, 1807-1813 (1999).
15. H. L. Jang *et al.*, Phase transformation from hydroxyapatite to the secondary bone mineral, whitlockite. *J. Mater. Chem. B.* **3**, 1342-1349 (2015).
